# Characterization of Chinese liquor aroma components during aging process and liquor age discrimination using gas chromatography combined with multivariable statistics

**DOI:** 10.1038/srep39671

**Published:** 2017-01-06

**Authors:** M. L. Xu, Y. Yu, H. S. Ramaswamy, S. M. Zhu

**Affiliations:** 1 College of Biosystems Engineering and Food Science, Zhejiang University, Hangzhou 310058, China; 2 Key Laboratory of Equipment and Informatization in Environment Controlled Agriculture, Ministry of Agriculture, Hangzhou 310058, China; 3 Department of Food Science, McGill University, St-Anne-de-Bellevue, QC H9X 3V9, Canada

## Abstract

Chinese liquor aroma components were characterized during the aging process using gas chromatography (GC). Principal component and cluster analysis (PCA, CA) were used to discriminate the Chinese liquor age which has a great economic value. Of a total of 21 major aroma components identified and quantified, 13 components which included several acids, alcohols, esters, aldehydes and furans decreased significantly in the first year of aging, maintained the same levels (p > 0.05) for next three years and decreased again (p < 0.05) in the fifth year. On the contrary, a significant increase was observed in propionic acid, furfural and phenylethanol. Ethyl lactate was found to be the most stable aroma component during aging process. Results of PCA and CA demonstrated that young liquor (fresh) and aged liquors were well separated from each other, which is in consistent with the evolution of aroma components along with the aging process. These findings provide a quantitative basis for discriminating the Chinese liquor age and a scientific basis for further research on elucidating the liquor aging process, and a possible tool to guard against counterfeit and defective products.

As one of the oldest alcoholic beverages, Chinese liquor possesses a long history of over 6000 years^1^. Due to the historical and cultural factors, this type of liquor plays a particular role in Chinese traditional culture, and it is now very popular in China and several other countries. Chinese liquor creates a sales income of over 60 billion dollars annually, with a consumption volume of over four million kiloliters^2^,^3^. Therefore, it takes up a significant position in the national economic development. Like some other alcoholic beverages such as whiskey and vodka, Chinese liquor aroma gets developed during the manufacturing process. Firstly, a mixture of several kinds of grains (sorghum is the main raw material) was milled and cooked, followed by fermentation in a specialized fermentor which coated with multiple layers of fermenting mud. Then the fermented product is distilled out with steam and saved as young liquor. The young liquor generally has unacceptable flavors which described as “pungent” or “coarse”, thus a prolonged aging process is needed to eliminate these negative attributes. Meanwhile, a well-balanced and mellow liquor aroma is formed adding economic value to the product. Therefore, aging plays an indispensable role in the process of producing high-quality Chinese liquor.

From quality perspective, aroma characteristic is the most important intrinsic factor making contributions to the overall quality of Chinese liquor, and is often used to discriminate liquors from different brands, regions and ages^4^. The overall aroma characteristic of Chinese liquor is generated by hundreds of chemical compounds balanced during the aging process; therefore, qualitative and quantitative analysis of liquor aroma components along with aging process is of great value for researchers and producers alike. During the past decade, aroma components of Chinese liquor have been studied extensively. Fan *et al*.^5^ used gas chromatography-olfactometry (GC-O) technique to investigate the aroma characteristic of Chinese liquor, and reported that 34 aroma components were important contributors for Chinese Chixiang-aroma-type liquor aroma, while 27 odorants were found to be important in light-aroma-type liquor by Gao *et al*.^6^ using the same method. The GC-O technique conduces to develop a better understanding of the aroma chemistry of Chinese liquor; however, it can be hard to provide concentration information since the odor threshold of aroma components is different for each component. To quantify aroma components in Chinese liquor, gas chromatography-mass spectrometry (GC-MS) has been widely used in a large number of researches^7^,^8^,^9^,^10^,^11^,^12^. GC-MS technique equipped with commercial databases performs well in quantitative analysis, especially when aroma components of the sample are rather complicated as in Chinese liquor. Meanwhile, since the water present in Chinese liquor can cause damage to MS, as an alternative, headspace solid-phase microextraction (HS-SPME) or other extraction methods are normally chosen. However, the extraction process can also be easily affected by extraction time, extraction temperature, solvent burden ratio and fiber type. So the accuracy of quantitative analysis by GC-MS could be questionable. In addition, few researchers have focused on changes in aroma components of Chinese liquor during the manufacturing process. Ding *et al*.^13^ investigated changes in volatile compounds of Chinese Luzhou-flavor liquor during the fermentation and distillation process using HS-SPME-GC-MS. Ma *et al*.^14^ reported variations in physicochemical properties of Chinese Fenjiu during storage using HS-SPME-GC-MS with three internal standard substance; however, these results cannot represent the actual concentration changes of aroma components since the correction factors of aroma components are much different from each other. Above all, very limited research has been conducted on the characterization and quantification of Chinese liquor aroma components along with the aging process using gas chromatography with direct injection method.

Since the market price of Chinese liquor is closely related to the aging time, so the authentication of liquor age is of great importance to protect consumers from being cheated. Principal component analysis (PCA) and cluster analysis (CA) based on chromatographic profiles are two multivariate statistical techniques which have been proved in several studies to discriminate and classify groups effectively^15^,^16^,^17^,^18^. The main objectives of this study were therefore: (1) to characterize Chinese liquor aroma components along with the aging process by accurate quantitative analysis, and to provide a theoretical basis for future researches which focus on the mechanism of aging process; (2) to explore the possibility of chromatographic profiles combined with multivariate statistic analysis to discriminate liquor age, and to fight against counterfeit and defective products.

## Results

### Identification and quantification analysis

**Figure 1:**
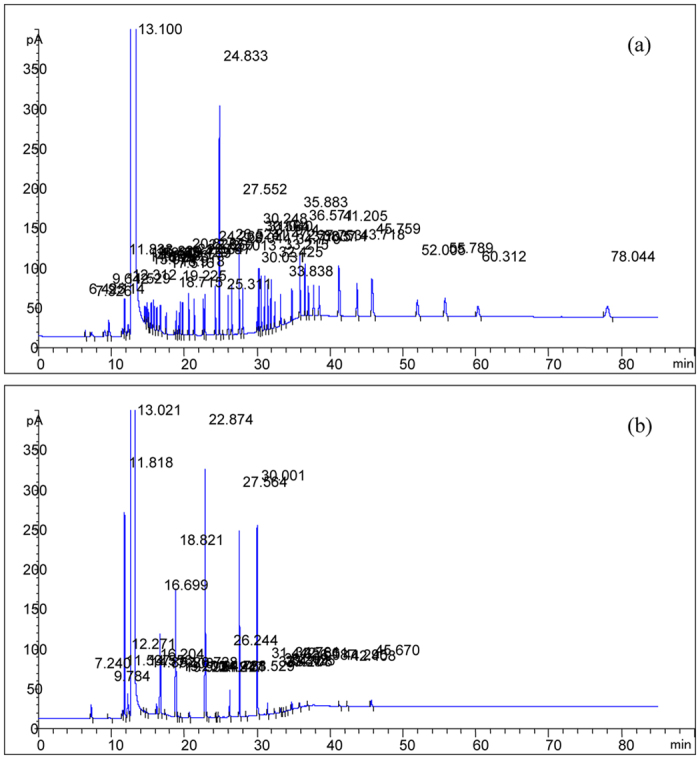
Typical Chromatogram map of standard solution (**a**) and liquor samples (**b**).

Typical GC chromatograms with direct injection of a mixed standard solution and liquor samples are shown in Fig. 1a and b. It demonstrated that 53 compounds of the mixed standard cocktail can be efficiently separated within 80 min, and the retention time of the last component was 78 min. For liquor samples, aroma components were well separated from each other within 46 min, and no more peaks were detected after that. All major aroma components of the Chinese liquor could be found in the mixed standard cocktail by comparing the retention times. Considering the chemical groups of acids, alcohols, esters, aldehydes and furans, 21 aroma components were identified.

As for the quantification analysis, detailed parameters of calibration curves and concentration ranges are summarized in Table 1. The correlation indexes (R^2^) acquired were from 0.9952 to 0.9995 for all components, which was in accordance with the requirement of GC analysis (R^2^ > 0.995). The slope of a regression curve stands for the sensitivity of GC analysis for corresponding component^19^. As shown in Table 1, the lowest sensitive quantification was 393.4 for acetal while the highest one was 4039.2 for phenylethyl alcohol. The detection limit was calculated as three times the baseline noise^19^ and ranged from 0.340 mg/L for n-butanol to 7.959 mg/L for ethyl acetate. Mass concentration ranges were determined according to preliminary experiment.

### Evolution of aroma components along with aging process

**Figure 2:**
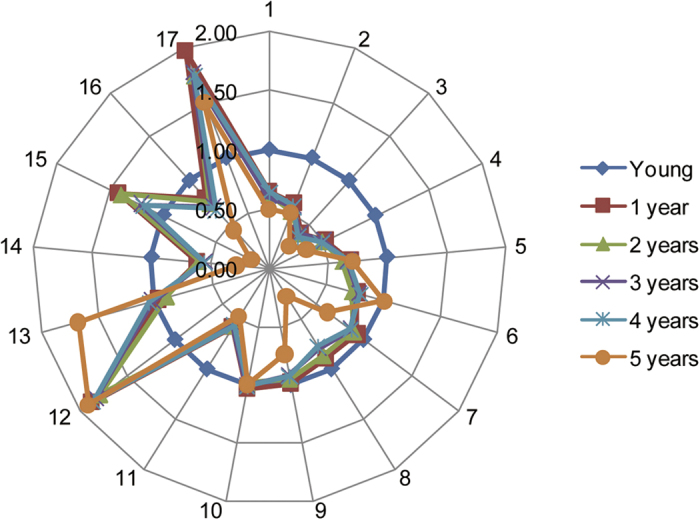
Radar map of aroma components. Acetaldehyde (1), Methanol (2), Ethyl acetate (3), Acetal (4), 2-Butanol (5), 1-Propanol (6), Isobutanol (7), n-Butanol (8), Isoamylol (9), Ethyl lactate (10), Acetic acid (11), Furfural (12), Propionic acid (13), Isobutyric acid (14), Butanoic acid (15), Isovaleric acid (16), Phenylethanol (17).

All aroma components of six liquor samples with different age (young liquor without storage and liquors stored for 1, 2, 3, 4 and 5 years) are shown in Table 2. One way ANOVA at the 5% significance level with Duncan method was used to compare statistical differences among six liquors. As can be seen from Table 2, volatile aroma components in six liquors were basically comprised of five chemical groups: acids, alcohols, esters, aldehydes and furans. The relatively low standard deviations obtained for most components confirmed the reliability and validity of the developed method. In order to visualize the general variation of aroma components during the aging process, a radar map based on concentrations of aroma components in liquor samples with different ages was used. As shown in Fig. 2, concentration of each aroma component in aged liquors was normalized by the one with young liquor. 17 components were selected since other 4 components were not detected in young liquor, and the amounts of these 4 components existed in aged liquors were also much lower as compared with other components.

#### Acids

Chinese liquor contains many acids, which are products of sugar oxidation or of ethanol fermentation during the liquor making process. Acids can have impact on sensory characteristics, contribute to color stability and increase antioxidant power^20^. Generally, acids are normally evaluated during the manufacturing of Chinese liquor to monitor the whole process and to ensure product quality. In this research, acids were investigated as possible markers of aging and also as an index for liquor age discrimination.

Five acids were detected: acetic acid, propionic acid, isobutyric acid, butanoic acid and isovaleric acid. As shown in Table 2, acetic acid was the most abundant components of this group with concentration of 1194 mg/L in young liquor, while the total amount of the other four acids was below 50 mg/L. As a matter of fact, total acid content in Chinese liquor is usually expressed as the concentration of acetic acid, as more than 90% of the acidity is contributed by it. In the one-year-aged liquor, the acetic acid content decreased significantly (p < 0.05) to 701 mg/L, however, no additional change was observed in 2–5 year old liquors. As for isobutyric acid, butanoic acid and isovaleric acid, a slight decline was continually observed during the aging process. As an exception, the content of propionic acid in five-year-aged liquor was found to be higher than other stored liquor groups (p < 0.05). The reduction in acids content may have been caused by some loss of volatile acids and esterification reactions between acids and alcohols during the aging process. In the aroma composition of Chinese liquor, acids are responsible for fruity, fatty as well as rancid notes. Short-chain acids which possess the aroma of sour and rancid could suppress and cover the other aroma in liquor, so the suitable acids concentration in liquor is of great importance and an increase in their content might lead to negative quality notes to liquor aroma^21^. From this point of view, the observed decline in acids content in Chinese liquor during the aging process could be considered conducive to the formation of a well-balanced aroma.

#### Alcohols

Alcohols usually generate from deaminization reactions of amino acids under anaerobic conditions and/or decarboxylation reactions of sugar under aerobic conditions during the fermentation process^22^. Sorghum used for producing Chinese liquor contains a high content of amino acids, which offers abundant precursor to alcohols, since these amino acids can be converted to alcohols by way of yeast fermentation^23^, and this is the main source of alcohols. Meanwhile, the reduction reaction of homologous aldehydes can also result in the increase of alcohols during the manufacturing process.

As can be seen from Table 2, 7 alcohols were detected by validated GC analysis method: methanol, 2-butanol, 1-propanol, isobutanol, n-butanol, isoamylol and phenylethanol. In this group, 1-propanol and isoamylol were the prominent alcohols since their concentrations found were 338, 637 and 800 mg/L, respectively, in the young liquor. After one year of storage, the content of 1-propanol decreased to 77% (p < 0.05), and maintained at this level during the following three years (p > 0.05). In the fifth year, however, the 1-propanol content increased rapidly and no significant difference was found between five-year-aged liquor and young liquor. Isobutanol and isoamylol seemed to be more stable during the first four years of aging (p > 0.05), however, reduction trends were observed in the fifth year, with the rate of 30% (isobutanol) and 21% (isoamylol) (p < 0.05). Though methanol exists in Chinese liquor with low content, the control of methanol level is still of great importance in consideration of its virulence. The content of methanol was 80 mg/L in young liquor, and then decreased to 46 mg/L after the first year, no significant change was observed after that. This level of methanol is within the commercial specification range for Chinese liquor (<400 mg/L). Small changes were also found in the content of 2-butanol, n-butanol and phenylethanol during aging process. It is recognized that there will be some decline in the alcohols content during the natural aging process of liquors, and this has been linked to the changes in the structure of water and alcohols molecules^24^.

#### Esters

Esters are generally generated by esterification reactions between organic acids and alcohols, especially ethanol, during the fermentation and aging processes. The activities of hydrolase and esterase exist in the raw material of Chinese liquor Daqu are powerful, thus catalyse synthesis of esters could be possible during the active fermentation process^25^. Since higher temperatures can lead to great losses in esters by ways of hydrolysis and volatilization, a relatively cooler temperature is needed during aging process. Ester group is the most affluent and crucial one exists in Chinese liquor, contributing to fruity, floral, and sweet aromas.

As listed in Table 2, 6 esters were detected: ethyl acetate, ethyl butyrate, ethyl hexanote, ethyl lactate, ethyl oenanthate and ethyl palmitate. Among those, ethyl acetate and ethyl lactate were the most prominent representatives, with concentrations of 1741 and 446 mg/L, respectively. Upon aging for one year, the concentration of ethyl acetate decreased to 694 mg/L (p < 0.05) and maintained at this level (p > 0.05) in the following three years, but then reduced by 29% in the fifth year (p < 0.05). Ethyl acetate is of great importance to the formation of distinctive aroma of distilled liquors, depending on its concentration^26^. Although ethyl acetate is usually regarded as sending out the smell of nail polish at high concentrations, it displays fruity aroma at low concentrations on the contrary^27^. It was observed that ethyl lactate was extremely stable during the aging process. Though the concentration fluctuated slightly in quantity, no significant difference was observed among six groups. Ethyl lactate is generally formed by lactic bacteria and plays an important role in stabilizing liquor flavor characteristics^28^. Ethyl butyrate, ethyl hexanote, ethyl oenanthate and ethyl palmitate had similar variation trends during aging process. As can be seen, long-chain esters were not found in young liquor, but formed in the first year of aging and decreased gradually in the following years. The reduction may have been caused by volatilization and permeation through the container with storage time. Overall, the decrease trend of total ester content found in this work (light-flavor Chinese liquor) was opposite with our previous research which focused on sauce-flavor Chinese liquor^29^. This could be mainly resulted from different liquor types, since the raw materials and manufacturing process of these two liquors are much different from each other.

#### Aldehydes and furans

In this group, 3 kinds of aroma components were detected: acetaldehyde, acetal and furfural. The variation trends of acetaldehyde and acetal were similar as shown in Table 2. Acetaldehyde and acetal decreased significantly in the first year with the rates of 36% and 47%, respectively. Again no significant change was observed in the middle three years, but after the five year of aging, further reductions were observed from 153 mg/L to 119 mg/L for acetaldehyde (p < 0.05) and from 255 mg/L to 183 mg/L for acetal (p < 0.05). On the contrary, the level of furfural increased from 19 mg/L to 36 mg/L (p < 0.05) during the first year and maintained at the same level (p > 0.05) in the following aging period.

Acetaldehyde is one kind of poisonous compound generates from oxidization of alcohol in food products^30^, which is often regarded as having sensorial properties of nut or overripe apples^31^. Acetaldehyde is also one of the important metabolites of ethanol in human body. First, ethanol is oxidized into acetaldehyde by ethanol dehydrogenase, and then acetaldehyde dehydrogenase oxidizes acetaldehyde into acetic acid, finally acetic acid will be transformed into carbon dioxide and water and excreted out of the body through the respiratory process. For people who don’t have enough acetaldehyde dehydrogenase, acetaldehyde can accumulate in the body causing headache, nausea and vomiting, which is one of the reasons that some people do not tolerate consumption of heavy liquors. Acetal is a very common colorless aroma component exists in distilled alcoholic beverages, with a pleasant smell at low concentrations making positive contributions to the flavor characteristics^32^. However, acetal at a higher concentration can be damaging to human health and can even cause death; the lethal dosage of acetal being about 500 mg/kg (body weight of consumer). At the level found which is about 250 mg/L, the acetal concentration levels are so far away from being lethal even if one were to consume the entire one liter volume liquor in one sitting. Furan can be formed by decomposition of carbohydrates under high temperature and dehydration of sugars through Maillard reaction, which occurs during fermentation and aging process and is usually considered as an aging marker^33^.

### Multivariate statistic analysis based on aroma components

#### Principal component analysis (PCA)

PCA is one kind of multivariate statistic analysis method that can be used for the analysis of a database pertaining to several inter-corrected dependent variables^34^, and it plays well in reducing the dimensionality of the database. PCA analysis involves the identification of different components: the first principal component contributes most to the total variance; similarly, each subsequent component has the largest contribution to the total variance with the orthorhombic restriction of previous components. Higher cumulative contribution rate of the variance is indicative of better inclusion of the original information^35^. Generally, the first and second principal component are selected and regarded as good representative of original information, when the accumulated variance exceed 80%.

**Figure 3:**
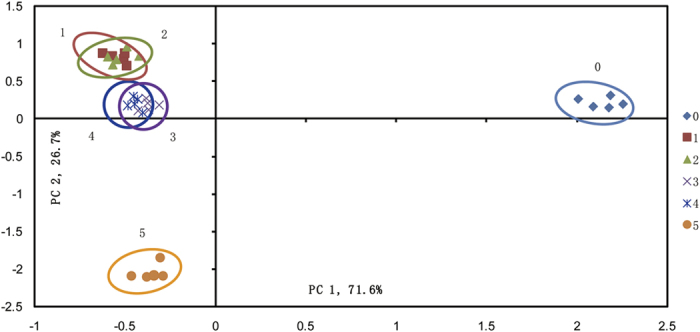
Scores plot of PCA based on aroma components. Young liquor (0), One-year-aged (1), Two-year-aged (2), Three-year-aged (3), Four-year-aged (4), Five-year-aged (5).

**Figure 4:**
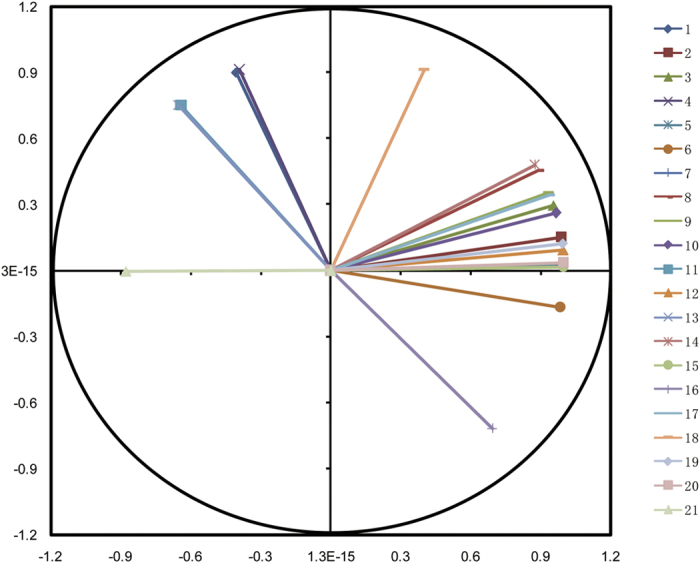
Loads plot of PCA based on aroma components. Acetaldehyde (1), Methanol (2), Ethyl acetate (3), Acetal (4), 2-Butanol (5), 1-Propanol (6), Ethyl butyrate (7), Isobutanol (8), n-Butanol (9), Isoamylol (10), Ethyl hexanoate (11), Ethyl lactate (12), Ethyl oenanthate (13), Acetic acid (14), Furfural (15), Propionic acid (16), Isobutyric acid (17), Butanoic acid (18), Isovaleric acid (19), Phenylethanol (20), Ethyl palmitate (21).

The scores plot and loads plot are as presented in Figs 3 and 4. Variables in Fig. 4 were numbered according to the list from Table 1. In this research, scores plot was used to locate liquor groups with different ages, and the loads plot was used to further research the contribution of specific aroma component to each principal component. As can be seen from Fig. 3, the first principal component (PC1) and second principal component (PC2) were taken as coordinate axes for the PCA analysis on samples, and it was noted that the linear combination of PC1 and PC2 explained 98.27% of the total variance of liquor samples. PC1 led to the separation of young liquor and aged liquors, since young liquor samples were grouped on the positive side of PC1 while aged liquors were located on the negative side. On the other hand, PC2 contributed to the differentiation of aged liquors stored for different years. Five-year-aged samples were located on the negative side of PC2 whereas other samples were located on the positive side. It should be noted that one-year-aged and two-year-aged samples were clustered as one category, this may has been caused by the similarity of aroma composition in those two liquors. Similar result was also observed between three-year-aged and four-year-aged liquors. More information can be found from the loads plot in Fig. 3b. As can be seen, almost all aroma components were positively related to PC1 and PC2, indicating a general decrease in concentrations of these aroma components as the liquor age increased. In addition, acetaldehyde (1), acetal (4), ethyl butyrate (7), ethyl hexanoate (11), ethyl oenanthate (13) and ethyl palmitate (21) were negatively related with PC1, meanwhile 1-propanol (6) and propionic acid (16) also had negative load values on PC2, which meant that these aroma components were positive related with liquor age. These results were in consistent with the evolution of aroma components along with aging process as presented before.

#### Cluster analysis (CA)

**Figure 5:**
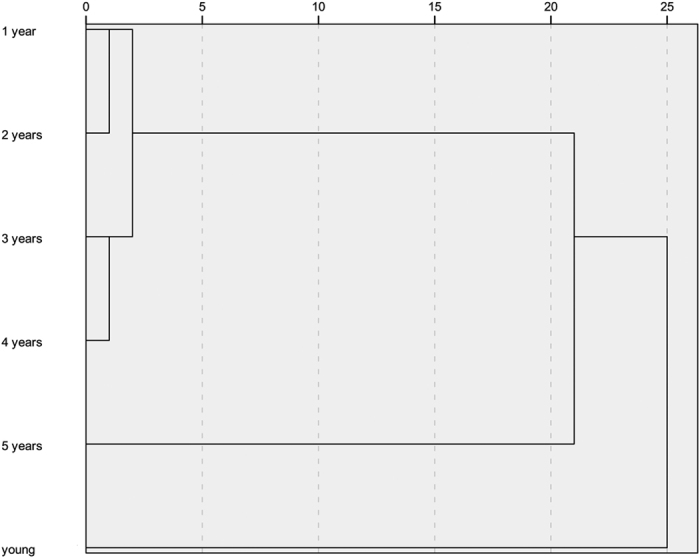
Results of CA based on aroma components.

In order to realize further separation among the six liquor samples, average values of 21 kinds of aroma components to six liquor samples were analyzed by CA using Euclidean distance. As shown in Fig. 5, all liquor samples were divided into two big groups. The first group consisted of aged liquors from one year to five years, while only young liquor was found in the second group, which meant young liquor and aged liquors were well separated from each other. To be more specific, in the first sub-cluster, one-year-aged liquor was much similar with two-year-aged one, while three-year-aged and four-year-aged liquors were clustered as one category. These results were mainly caused by the similar composition of aroma components in liquors, which was also observed in PCA results. Furthermore, there must be much difference between five-year-aged liquor and other four aged liquors, since the former one was found to be existed as an independent category. These findings can be verified by the evolution of aroma components as presented before, since significant changes were observed in the fifth year during aging process. It can be concluded that cluster analysis based on aroma components performed well in the discrimination of Chinese liquor with different ages.

## Conclusions

A gas chromatography analysis with direct injection technique was used for the identification and quantification of Chinese liquor aroma components along with the aging process. Based on the study, 21 aroma components were found to be important in the aroma profile of Chinese liquor. Of these, 13 aroma components decreased significantly with one year of aging, but maintained at the same level (p > 0.05) in the next three years, and decreased again (p < 0.05) after the five year aging. On the other hand, a significant increase was observed in propionic acid, furfural and phenylethanol. Ethyl lactate was found to be more stable than other aroma components during aging process. PCA and CA based on GC results were used to discriminate Chinese liquor with different ages based on the aroma components. Young liquor and aged liquors were well separated from each other, results of which were consistent with the evolution of aroma components during the aging process. The study contributes to a further understanding of the role of aroma components developed during the aging of Chinese liquor. A better comprehension of this knowledge will aid to distinguish the characteristic aroma of Chinese liquor with different liquor age and identify counterfeit and defective products.

## Materials and Methods

### Chinese liquor samples and chemicals

Chinese liquor “*Junchang*” (light-style) was provided by a local liquor factory in Sichuan province, and stored at room temperature before use. *Junchang* is a popular local brand of Chinese liquor produced by Junchang Liquor Factory, which was established more than ten years ago with an annual output of over 300 kiloliters in recent years. It is not as famous as Moutai and other major brands and is mostly sold locally. Young liquor without storage and liquors stored at the factory for 1, 2, 3, 4 and 5 years, were analyzed. One mL aliquot of liquor samples was injected into a 2 mL autosampler vials without any pretreatment and then placed at room temperature before use. A solution containing mixed standards (Donglilong Information Technology Co. Ltd., Lanzhou, China), which contained 53 aroma components, was specially prepared for Chinese liquor gas chromatographic (GC) analysis (qualitative and quantitative analysis). The mixed standard solution was first diluted using ethanol with to give solution to ethanol ratio of 1:0, 1:1, 1:3, 1:7, 1:15, 1:31, 1:63 and 1:99, respectively, and then 1 mL of the diluted solution was injected into a 2 mL autosampler vials. This series of diluted solution was used for the protraction of standard curves.

### Gas chromatography analysis

Gas chromatography analysis was performed using an Agilent 7890A gas chromatography (GC) unit equipped with a flame ionization detector (FID). The column carrier gas was nitrogen at constant flow rate of 1 mL/min. Liquor samples were analyzed on a LZP-950 column (50 m × 0.32 mm i.d., 1.0 μm film thickness), which was also special for Chinese liquor gas chromatographic analysis. A measured volume (1 μL) of test sample was injected into the GC, and the split ratio was 1:1. The oven temperature was held at 65 °C for 8 min, then raised to 200 °C at a rate of 5 °C/min, and held at 200 °C for 50 min; injector and detector temperature were 230 °C and 250 °C respectively.

The identification of aroma components in liquor samples was made by comparing the retention times with corresponding components in the mixed standard solution. The quantification of aroma components was made based on calibration curves which were obtained with the mixed standard solution under the same chromatographic conditions as those of the liquor samples. All liquor samples were analyzed by direct injection method, calculated in five replicates and the values were averaged.

### Statistical analysis

Data of all groups obtained from GC analysis were initially processed by One Way ANOVA at the 5% significance level using IBM SPSS Statistics 21.0 to compare the differences statistically. Then multivariate statistical analysis was used to discriminate liquors with different ages, and to establish a correlation between aroma components and liquor age. In this research, PCA was applied to extract the first and second principal components from aroma components data and explore the possibility of discriminating differences between various samples. CA is an unsupervised clustering procedure based on the similarity or distances among observations^36^. PCA and CA analysis were also conducted with IBM SPSS Statistics 21.0.

## Additional Information

**How to cite this article**: Xu, M. L. *et al*. Characterization of Chinese liquor aroma components during aging process and liquor age discrimination using gas chromatography combined with multivariable statistics. *Sci. Rep.* **7**, 39671; doi: 10.1038/srep39671 (2017).

**Publisher's note:** Springer Nature remains neutral with regard to jurisdictional claims in published maps and institutional affiliations.

## Figures and Tables

**Table 1:** Calibration curves for the quantification analysis of liquor aroma components.

Number	Components	Linearity range (mg/L)	Slope (a)	Intercept (b)	LOD (mg/L)	R^2^ (n = 8)
1	Acetaldehyde	6.06–606	505.4	15.9	2.097	0.9962
2	Methanol	3.18–318	989.9	24.3	2.455	0.9959
3	Ethyl acetate	23.25–2325	909.7	72.4	7.959	0.9958
4	Acetal	6.46–646	393.4	34.1	4.334	0.9963
5	2-Butanol	3.58–358	1533.7	31.4	2.047	0.9988
6	1-Propanol	4.26–426	1904.4	45.0	2.363	0.9976
7	Ethyl butyrate	1.27–127	1494.0	11.2	0.750	0.9981
8	Isobutanol	10.92–1092	1530.8	27.5	1.796	0.9970
9	n-Butanol	3.87–387	2174.2	7.4	0.340	0.9993
10	Isoamylol	10.38–1038	2051.3	44.4	2.164	0.9979
11	Ethyl hexanoate	2.98–298	1815.4	109.5	0.603	0.9972
12	Ethyl lactate	9.17–917	2091.5	80.9	1.547	0.9981
13	Ethyl oenanthate	3.64–364	2126.5	23.2	1.091	0.9983
14	Acetic acid	15.48–1548	935.3	14.0	1.497	0.9952
15	Furfural	5.41–541	1501.8	30.8	2.051	0.9978
16	Propionic acid	3.88–388	1330.1	4.6	0.346	0.9972
17	Isobutyric acid	1.40–140	1756.0	0.3	0.342	0.9995
18	Butanoic acid	1.20–120	1631.3	7.3	0.179	0.9970
19	Isovaleric acid	1.36–136	3802.7	15.0	0.394	0.9988
20	Phenylethanol	2.10–210	4039.2	46.1	1.141	0.9979
21	Ethyl palmitate	4.60–460	2517.0	49.0	1.947	0.9959

Regression equation: y = ax + b, where y is the peak area and x is the concentration mg/L.

LOD: limit of detection, calculated by three times the ratio of signal/noise.

R^2^: correlation coefficient.

**Table 2:** Quantitative analysis results of Chinese liquor aroma components with different age.

Number	Components	Young	One year	Two years	Three years	Four years	Five years
1	Acetaldehyde	237.81 ± 11.87a	152.99 ± 12.67b	148.14 ± 6.34b	144.73 ± 13.72b	153.67 ± 5.76b	118.96 ± 4.53c
2	Methanol	79.79 ± 3.46a	46.75 ± 2.06b	41.09 ± 1.66b	44.62 ± 3.97b	44.68 ± 2.35b	39.86 ± 1.11b
3	Ethyl acetate	1741.48 ± 87.52a	694.23 ± 36.17b	677.85 ± 44.60b	692.20 ± 164.33b	627.80 ± 32.33b	446.29 ± 20.55c
4	Acetal	519.72 ± 30.60a	275.31 ± 22.76b	260.37 ± 11.09b	267.91 ± 23.17b	255.42 ± 18.14b	183.57 ± 6.46c
5	2-Butanol	29.97 ± 1.35a	20.54 ± 2.54b	18.74 ± 1.44b	20.02 ± 1.95b	20.18 ± 1.90b	21.16 ± 1.69b
6	1-Propanol	338.84 ± 16.54a	261.24 ± 20.35b	247.04 ± 7.04b	267.68 ± 23.10b	270.81 ± 13.65b	341.57 ± 10.96a
7	Ethyl butyrate	ND	4.28 ± 0.53a	4.00 ± 0.21a	3.05 ± 0.59a	2.27 ± 0.19b	ND
8	Isobutanol	637.67 ± 39.72a	593.72 ± 48.15a	573.37 ± 29.01a	551.78 ± 39.16a	549.03 ± 35.42a	390.93 ± 22.71b
9	n-Butanol	13.84 ± 1.73a	12.53 ± 1.79a	12.09 ± 1.38a	11.08 ± 1.14a	10.71 ± 1.43a	3.85 ± 0.62b
10	Isoamylol	800.53 ± 41.79a	793.02 ± 38.19a	766.17 ± 19.15a	740.56 ± 52.14a	744.52 ± 44.75a	588.39 ± 18.53b
11	Ethyl hexanoate	ND	52.82 ± 4.69a	50.16 ± 2.15a	35.88 ± 5.23b	31.42 ± 0.64b	ND
12	Ethyl lactate	446.38 ± 30.40a	465.10 ± 31.16a	445.73 ± 27.98a	450.21 ± 19.36a	454.67 ± 31.46a	443.85 ± 23.94a
13	Ethyl oenanthate	ND	14.43 ± 1.62a	13.82 ± 0.51a	10.11 ± 1.23a	9.45 ± 0.30a	ND
14	Acetic acid	1194.00 ± 91.82a	701.73 ± 40.19b	688.29 ± 37.69b	673.71 ± 44.96b	676.13 ± 38.74b	570.52 ± 30.31c
15	Furfural	19.17 ± 1.09b	36.05 ± 2.56a	34.17 ± 0.57a	35.10 ± 2.09a	35.71 ± 2.33a	36.59 ± 1.07a
16	Propionic acid	7.83 ± 0.57b	7.61 ± 0.41b	7.00 ± 0.10b	8.03 ± 0.19b	8.24 ± 0.48b	13.07 ± 0.46a
17	Isobutyric acid	14.95 ± 1.92a	9.18 ± 0.60b	8.90 ± 0.20b	8.22 ± 0.29b	8.22 ± 0.66b	4.13 ± 0.11c
18	Butanoic acid	20.87 ± 1.56a	29.69 ± 2.01a	29.07 ± 0.71a	25.22 ± 0.72a	24.72 ± 1.93a	3.49 ± 0.28b
19	Isovaleric acid	3.62 ± 0.26a	2.87 ± 0.18a	2.68 ± 0.04a	2.51 ± 0.07a	2.43 ± 0.16a	1.58 ± 0.05b
20	Phenylethanol	5.90 ± 0.44b	11.59 ± 0.68a	10.30 ± 0.20a	10.42 ± 0.25a	10.32 ± 0.73a	8.85 ± 0.29a
21	Ethyl palmitate	ND	13.09 ± 0.95a	15.24 ± 1.09a	14.69 ± 0.57a	15.31 ± 1.10a	10.74 ± 0.68b

All values are expressed as means (mg/L) ± standard deviation (SD).

Different letters indicate significant differences (p < 0.05).

ND: Not detected.

## References

[b1] Feng Y. . Chemical Analysis of the Chinese Liquor Luzhoulaojiao by Comprehensive Two-Dimensional Gas Chromatography/Time-of-Flight Mass Spectrometry. Sci Rep. 5, 9553, doi: 10.1038/srep09553 (2015). 25857434 PMC4392506

[b2] Fan W. & Qian M. C. Characterization of aroma compounds of Chinese “*Wuliangye*” and “*Jiannanchun*” liquors by aroma extract dilution analysis. J. Agric. Food Chem. 54(7), 2695–2704 (2006). 16569063 10.1021/jf052635t

[b3] Fan W. & Qian M. C. Headspace solid phase microextraction and gas chromatography-olfactometry dilution analysis of young and aged Chinese “Yanghe Daqu” liquors. J. Agric. Food Chem. 53(20), 7931–7938 (2005). 16190652 10.1021/jf051011k

[b4] Xiao Z. . Characterization of aroma compounds of Chinese famous liquors by gas chromatography–mass spectrometry and flash GC electronic-nose. J. Chromatogr. B. 945, 92–100 (2014). 10.1016/j.jchromb.2013.11.032 24333641

[b5] Fan H., Fan W. & Xu Y. Characterization of Key Odorants in Chinese Chixiang Aroma-Type Liquor by Gas Chromatography–Olfactometry, Quantitative Measurements, Aroma Recombination, and Omission Studies. J. Agric. Food Chem. 63(14), 3660–3668 (2015). 25797496 10.1021/jf506238f

[b6] Gao W., Fan W. & Xu Y. Characterization of the key odorants in light aroma type Chinese liquor by gas chromatography–olfactometry, quantitative measurements, aroma recombination, and omission studies. J. Agric. Food Chem. 62(25), 5796–5804 (2014). 24909925 10.1021/jf501214c

[b7] Zheng J., Liang R., Wu C., Zhou R. & Liao X. Discrimination of different kinds of Luzhou-flavor raw liquors based on their volatile features. Food Res. Int. 56, 77–84 (2014).

[b8] Du L., He T., Li W., Wang R. & Xiao D. Analysis of volatile compounds in Chinese Laobaigan liquor using headspace solid-phase microextraction coupled with GC-MS. Anal. Methods. 7(5), 1906–1913 (2015).

[b9] Fan W., Shen H. & Xu Y. Quantification of volatile compounds in Chinese soy sauce aroma type liquor by stir bar sorptive extraction and gas chromatography-mass spectrometry. J. Sci. Food Agric. 91(7), 1187–1198 (2011). 21384368 10.1002/jsfa.4294

[b10] Wang L. . Identification and Aroma Impact of Volatile Terpenes in Moutai Liquor. Int. J. Food Prop. 19(6), 1335–1352 (2016).

[b11] Zhang R., Wu Q. & Xu Y. Aroma characteristics of Moutai-flavour liquor produced with Bacillus licheniformis by solid‐state fermentation. Lett. Appl. Microbiol. 57(1), 11–18 (2013). 23594087 10.1111/lam.12087

[b12] Wang P. P., Li Z., Qi T. T., Li X. J. & Pan S. Y. Development of a method for identification and accurate quantitation of aroma compounds in Chinese Daohuaxiang liquors based on SPME using a sol–gel fibre. Food chem. 169, 230–240 (2015). 25236221 10.1016/j.foodchem.2014.07.150

[b13] Ding X., Wu C., Huang J. & Zhou R. Changes in Volatile Compounds of Chinese Luzhou‐Flavor Liquor during the Fermentation and Distillation Process. J. Food Sci. 80 **(11)**, 2373–2381(2015). 10.1111/1750-3841.13072 26444440

[b14] Ma Y. . Variations in physicochemical properties of Chinese Fenjiu during storage and high-gravity technology of liquor aging. Int. J. Food Prop. 17(4), 923–936 (2014).

[b15] Xiao Z., Yu D., Niu Y., Ma N. & Zhu J. Characterization of different aroma-types of Chinese liquors based on their aroma profile by gas chromatography–mass spectrometry and sensory evaluation. Flavour Frag. J. (2016).

[b16] Serrano-Lourido D., Saurina J., Hernández-Cassou S. & Checa A. Classification and characterisation of Spanish red wines according to their appellation of origin based on chromatographic profiles and chemometric data analysis. Food Chem. 135(3), 1425–1431 (2012). 22953876 10.1016/j.foodchem.2012.06.010

[b17] Consonni R., Cagliani L. R., Guantieri V. & Simonato B. Identification of metabolic content of selected Amarone wine. Food Chem. 129(2), 693–699(2011). 10.1016/j.foodchem.2011.05.008 30634288

[b18] Ding X., Wu C., Huang J. & Zhou R. Characterization of interphase volatile compounds in Chinese Luzhou-flavor liquor fermentation cellar analyzed by head space-solid phase micro extraction coupled with gas chromatography mass spectrometry (HS-SPME/GC/MS). LWT-Food Sci. Technol. 66, 124–133 (2016).

[b19] López-Vázquez C., Bollaín M. H., Berstsch K. & Orriols I. Fast determination of principal volatile compounds in distilled spirits. Food Control 21(11), 1436–1441 (2010).

[b20] De Villiers A., Vanhoenacker G., Majek P. & Sandra P. Determination of anthocyanins in wine by direct injection liquid chromatography–diode array detection–mass spectrometry and classification of wines using discriminant analysis. J. Chromatogr. A 1054(1), 195–204 (2004). 15553144

[b21] Bordiga M. . Characterization of Muscat wines aroma evolution using comprehensive gas chromatography followed by a post-analytic approach to 2D contour plots comparison. Food Chem. 140(1), 57–67 (2013). 23578615 10.1016/j.foodchem.2013.02.051

[b22] Russell I. Understanding yeast fundamentals. The alcohol textbook 4, 531–537(2003).

[b23] Wondra M. & Berovic M. Analyses of aroma components of Chardonnay wine fermented by different yeast strains. Food Technol. Biotechnol. 39(2), 141–148 (2001).

[b24] Nishimura K. & Matsuyama R. Maturation and maturation chemistry. The science and technology of whiskies. 235–263 (1989).

[b25] Fan W. & Qian M. C. Identification of aroma compounds in Chinese ‘Yanghe Daqu’liquor by normal phase chromatography fractionation followed by gas chromatography [sol] olfactometry. Flavour Frag. J. 21(2), 333–342 (2006).

[b26] Malcata F. X. Macedo A. C. & Silva M. L. O. Steam distilled spirits from fermented grape pomace: Review. Food Sci. Technol Int. 6(4), 285–300 (2000).

[b27] Cortés S., Gil M. L. & Fernández E. Volatile composition of traditional and industrial Orujo spirits. Food Control 16(4), 383–388 (2005).

[b28] Apostolopoulou A. A., Flouros A. I., Demertzis P. G. & Akrida-Demertzi K. Differences in concentration of principal volatile constituents in traditional Greek distillates. Food Control 16(2), 157–164 (2005).

[b29] Zhu S. M. . Effect of high pressure treatment on the aging characteristics of Chinese liquor as evaluated by electronic nose and chemical analysis. Sci. Rep. 6, 30273, doi: 10.1038/srep30273 (2016). 27484292 PMC4971502

[b30] Silva M. L. & Malcata F. X. Relationships between storage conditions of grape pomace and volatile composition of spirits obtained therefrom. Am. J. Enol. Vitic. 49(1), 56–64 (1998).

[b31] Anli R. E., Vural N. & Gucer Y. Determination of the principal volatile compounds of Turkish Raki. J. Inst. Brew. 113(3), 302–309 (2007).

[b32] Cabaroglu T. & Yilmaztekin M. Methanol and major volatile compounds of Turkish Raki and effect of distillate source. J. Inst. Brew. 117(1), 98–105 (2011).

[b33] Alañón M. E., Rubio H., Díaz-Maroto M. C. & Pérez-Coello M. S. (2010) Monosaccharide anhydrides, new markers of toasted oak wood used for ageing wines and distillates. Food Chem. 119(2), 505–512 (2010).

[b34] Abdi H. & Williams L. J. Principal component analysis. Comput. Stat. 2(4), 433–459 (2010).

[b35] Qiu S., Wang J. & Gao L. Qualification and quantisation of processed strawberry juice based on electronic nose and tongue. LWT-Food Sci. Technol. 60(1), 115–123 (2015).

[b36] Huang J. Y., Guo X. P., Qiu Y. B. & Chen Z. Y. Cluster and discriminant analysis of electrochemical noise data. Electrochimi. Acta. 53(2), 680–687 (2007).

